# Outcomes of Women with Preeclampsia and Eclampsia Admitted in the Intensive Care Unit at a Tertiary Care Hospital in Mogadishu, Somalia

**DOI:** 10.1155/2023/6641434

**Published:** 2023-11-10

**Authors:** Nasra Mohamud Hilowle, Said Abdirahman Ahmed, Khadija Yusuf Ali, Ercan Altinel, Mohamud Mire Waberi, Mohamed Sheikh Hassan, Diyar Köprülü, Abdijalil Abdullahi Ali, Mohamed Omar Hassan

**Affiliations:** ^1^Department of Anesthesiology and Reanimation, Mogadishu Somalia Turkish Training and Research Hospital, Somalia; ^2^Department of Cardiology, Mogadishu Somalia Turkish Training and Research Hospital, Somalia; ^3^Department of Obstetrics and Gynecology, Mogadishu Somalia Turkish Training and Research Hospital, Somalia; ^4^Department of Neurology, Mogadishu Somalia Turkish Training and Research Hospital, Somalia; ^5^Department of Cardiovascular Surgery, Mogadishu Somalia Turkish Training and Research Hospital, Somalia

## Abstract

Intensive care for a hypertensive mother with preeclampsia or eclampsia is crucial for both maternal and neonatal outcomes. This study highlights the level of morbidity and mortality among women with preeclampsia and eclampsia admitted to the intensive care unit. *Methods*. This retrospective study was conducted in Mogadishu, Somalia, at the Mogadishu Somali Türkiye Training and Research Hospital from February 2019 to July 2022. The study focused on the different complications, managements, and final outcomes of preeclampsia and eclampsia mothers admitted to the intensive care unit. The data was retrieved from the electronic records of patients admitted to the intensive care unit. *Results*. During our study period, a total of 237 patients were identified as having preeclampsia/eclampsia, of whom 71 required intensive care admission. The mean age of the studied patients was 25 ± 6 years. The most common reason for being taken to the intensive care unit (ICU) was having a seizure (*n* = 33, 46.5%), followed by having very high blood pressure (*n* = 20, 28.2%), and being confused (*n* = 18, 25.3%). Peripartum infection was the most common maternal complication during ICU admission (66.7%), followed by cardiac-related arrhythmia (66.7%), postpartum bleeding (48%), acute kidney injury (18.4%), HELLP syndrome (16.4%), severe anemia (9.6%), and stroke (8.7%). Among patients, 65 (91.5%) needed mechanical ventilation. About 11.1% of these patients died during hospitalization. There were associations between mortality and some complications, particularly acute kidney injury (*p* value less than 0.02) and peripartum infection (*p* value less than 0.003). *Conclusion*. Hypertensive disease of pregnancy (preeclampsia/eclampsia) requiring intensive care unit admission has a very high morbidity and mortality rate.

## 1. Introduction

In pregnancy, hypertension is diagnosed when systolic blood pressure (SBP) is 140 mmHg and/or diastolic blood pressure (DBP) is 90 mmHg; it must be confirmed, preferably on two distinct occasions or at least 15 minutes apart in severe hypertension (i.e., 160/110 mmHg in the obstetric literature) [1]. Preeclampsia, also known as pregnancy-induced hypertension, is characterized by high blood pressure and proteinuria after 20 weeks [2] and HELLP (hemolysis, increased liver enzymes, and low platelet count), thrombocytopenia, decreased liver function, renal insufficiency, pulmonary edema, or a cerebral or visual disorder in the absence of proteinuria [3]. Eclampsia, on the other hand, is very similar to preeclampsia in terms of definition but includes a presentation with either convulsions or unconsciousness. Nulliparity, prior preeclampsia, a family history of preeclampsia, and increased maternal age are all risk factors for the development of preeclampsia. Preexisting medical conditions such as hypertension, diabetes, renal illness, and antiphospholipid syndrome raise the maternal risk of preeclampsia [4].

Clinical manifestations of preeclampsia were categorized as mild, moderate, or severe based on BP and also classified as early or late preeclampsia according to the gestational age of onset. The amount of proteinuria was deemed ineffective in determining severity [5]. Preeclampsia and gestational hypertension (hypertension after 20 weeks of pregnancy) are the major causes of maternal and neonatal morbidity and mortality globally [6]. One of the key causes would seem to be the absence of efficient screening and management policies.

Although PE-related mortality is rare in nations with abundant resources, morbidity is high and contributes significantly to hospital admissions during pregnancy [7]. In developed nations, the incidence is roughly 3.4%, whereas in developing countries, it ranges from 1.8% to 16.7% [8].

In the study conducted in the Somali region, there was a higher prevalence of hypertensive disorders of pregnancy (19.1%) [9].

According to the Somalia Health Demographic Survey report for 2020, the maternal mortality rate (MMR) in Somalia is high, at 692 per 100,000 live births [10]. One major contributing factor to such deaths is the lack of visits to maternal-child health centers (MCH) and getting any prenatal care (ANC).

Preeclampsia affects not only maternal morbidity and mortality but also neonatal morbidity and mortality. It can result in fetal growth restriction due to oligohydramnios, premature birth, low birth weight, severe birth asphyxia, stillbirth, and intrapartum death. One study found that birth weight is reduced by 5% because of preeclampsia. Birth weight is 23% lower than anticipated in cases of early-onset disease and severe preeclampsia [11]. The risks of high systolic blood pressure, especially when combined with a low platelet count, appear to be greatly underestimated in terms of intracerebral hemorrhage [12]. Preeclampsia with postpartum hemorrhage, eclampsia, kidney failure, and pulmonary edema/heart failure were some causes for admission to the ICU. Other causes included preeclampsia with dysregulated hypertension or preeclampsia combined with HELLP syndrome [13]. The primary objective of our study was to determine the morbidity and mortality associated with hypertensive pregnant mothers admitted to the intensive care unit.

## 2. Methodology and Materials

### 2.1. Study Population

A total of 71 patients were admitted to the ICU with hypertensive disease of pregnancy (preeclampsia/eclampsia) from February 2019 to July 2022. The characteristics that were addressed included age, gravity, gestational age and parity, mode of delivery, liver function test, thyroid function test, duration of admission, complications during admission, and finally their outcome.

### 2.2. Case Definitions

*Gestational hypertension* was defined as a systolic BP of at least 140 mm Hg and/or a diastolic BP of at least 90 mm Hg on two occasions at least 6 hours apart after the 20th week of gestation in normotensive women before pregnancy [14]. When gestational hypertension ≥160 mmHg systolic and/or ≥110 mmHg diastolic, it is termed as preeclampsia with a severe feature [15]. *Preeclampsia* was defined as based on the American College of Obstetrics and Gynecology (ACOG)-issued practice bulletin of 2019, the 2013 Task Force, and the guidelines prompted by the International Society for the Study of Hypertension in Pregnancy (ISSHP). By meeting three criteria: pregnancy >20 weeks, proteinuria (2+ on dipstick or > 300 mg/24 h), and arterial hypertension ≥140/90 mm Hg. *Eclampsia* was defined as the presence of one or more widespread convulsions and/or coma in the presence of preeclampsia and the absence of other neurologic disorders [16].

### 2.3. Statistical Analysis

The data were analyzed using the Statistical Package for Social Sciences (SPSS) software version 26 which was used to conduct the statistical analysis. Descriptive data analysis and Pearson correlation were mainly used in this study. *p* < 0.05 was considered statistically significant.

### 2.4. Ethical Consideration

Due to the fact that our hospital is a research hospital, a general informed consent is obtained from every patient admitted to obtain their data for retrospective research purposes from the hospital medical records, and this study did not disclose any personal information. The study was approved by the research ethics committee of our hospital (Ethics Protocol No: MSTH/7268). The study was performed in line with the principles of the Declaration of Helsinki. An early preprint was uploaded of this article which was updated recently to further improve the quality of our paper.

## 3. Result

**Figure 1:**
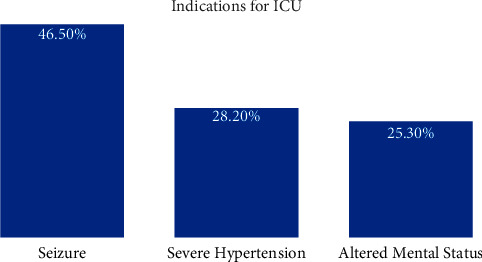
Demonstrates the main indications of intensive care unit admission.

During our study period, a total of 237 patients were identified as having preeclampsia/eclampsia, of whom 71 required intensive care admission which is about 2% of the total number who required ICU admission at the time which is 3714. The mean age was 25 ± 6 years, with a range of 15–40 years old. Considering the participants' comorbidities, 38 out of 71 had comorbid conditions, including history of gestational hypertension, 26 (36.6%), a history of previous preeclampsia 8 (11.2%) and history of gestational diabetes 4 (5.6%), while 33% of the patients did not have any recognized comorbidity. The primary reason for intensive care unit (ICU) admission was convulsion (*n* = 33, 46.5%), followed by severely elevated blood pressure (*n* = 20, 28.2%) and disorientation/confusion (*n* = 18, 25.3%) (Figure 1).

The clinical characteristics of these patients, as presented in (Table 1), demonstrated that those in their 3^rd^ trimester were the most common ones who had preeclampsia. The majority of our participants, on the other hand, were primigravida (*n* = 53, 74.6%) and nulliparous, *n* = (50, 70%).

Peripartum infection was the most common maternal complication during ICU admission 50(66.7%), followed by cardiac-related arrhythmia 50 (66.7%), bleeding 36 (48%), acute kidney injury 18 (25.4%), HELLP syndrome 16 (22.5%), severe anemia 9 (12.6%), and stroke 8 (10.7%) (Table 2). Even though less than 10% of patients were admitted with severe anemia necessitating a transfusion, 51 (71.8%) received a transfusion for other reasons, such as severe bleeding or dialysis. 10 (14.1%) needed hemodialysis due to acute kidney injury. On the other hand, 65 (91.5%) needed mechanical ventilation (Table 3). For delivery, 70 (99%) had a cesarean section, while 1 (1%) had a vaginal delivery.

The majority of the deliveries (75.7%) were performed under general anesthesia, while the remainder of the deliveries (24.3%) was performed under spinal anesthesia. Since the number of patients who needed mechanical ventilation was high, the outcome of the patients was not good. About 11.1% of patients died during hospitalization. There was a strong association between death and some complications, particularly acute kidney injury (*p* value less than 0.02) and peripartum infection (*p* value less than 0.003).

## 4. Discussion

Hypertensive disorders of pregnancy impact 10% of pregnant women globally [17]. Chronic (preexisting) hypertension, gestational hypertension, and preeclampsia cause 14% of maternal fatalities globally, and the rate of recurrent eclampsia in subsequent pregnancies is believed to be around 2% [16, 18]. Preeclampsia is the most prevalent medical complication of pregnancy and major cause of maternal, fetal, and newborn morbidity and mortality [19]. It complicates 5 to 8% of all pregnancies, which equates to 8.5 million cases every year worldwide [20].

Clark et al. [21] reported 95 maternal fatalities in 1,461,270 pregnancies after evaluating all maternal deaths in the United States among nearly 1.5 million deliveries in 124 hospitals (6.5 per 100,000 pregnancies). The average maternal age of the women who died was 29 years (range, 13–42). In our study, the average age was more than 20 years. These complications can be classified into obstetric and nonobstetric complications. Obstetric problems caused by preeclampsia include intrauterine growth restriction (IUGR), intrauterine fetal death (IUFD), early delivery, HELLP syndrome, and eclampsia. On the other hand, heart failure, peripartum cardiomyopathy, pulmonary edema, posterior reversible encephalopathy syndrome, stroke, renal failure, acute kidney injury, future risk of end-stage renal disease, liver failure, hepatic rupture, and coagulopathy are nonobstetric complications in preeclampsia [22].

Preeclampsia is a major risk for both early and late complications affecting both the mother and the infant. These complications include pulmonary thromboembolism, amniotic fluid embolism, obstetric hemorrhage, sepsis, acute renal failure, postpartum cardiomyopathy, and neurologic complications including cerebral venous thrombosis and intracerebral hemorrhage [12, 23]. In this study, the death rate was very high among those with severe infections and acute renal failure.

In addition to this early complication, women with preeclampsia have a high risk of developing late complications such as chronic hypertension (3.7 times higher risk of developing hypertension later in life), coronary heart disease (2.2 times increased risk), and stroke (1.8 times higher risk) [24]. Despite significant efforts to identify predicted risk factors for maternal and prenatal outcomes, preeclampsia typically has an unexpected prognosis, with maternal and fetal health deteriorating within hours [25]. Early suspicion and diagnosis, as well as early treatment, are important for good results. Early management includes fluid balance, pressure control with intravenous (IV) antihypertensive agents balanced with the risk of acute fetal compromise, and MgSO4 [26]. In some patients with ongoing oliguria, pulmonary edema, or other complicating medical issues, intensive monitoring via central venous pressure may be appropriate. The goal of antihypertensive medication is to keep systolic blood pressure (BP) between 140 and 155 mmHg and diastolic blood pressure (BP) between 90 and 105 mmHg [14]. Even though both labetalol and nifedipine are as effective as hydralazine in the acute setting and are much less likely to cause abrupt, profound hypotension, no single antihypertensive has been shown to be superior to another [27, 28]. By ACOG recommendation, intravenous (IV) hydralazine, labetalol, and oral nifedipine are for acute hypertensive urgency, and oral calcium channel blockers and labetalol are recommended for long-term control [29].

Although delivery is the definitive treatment for preeclampsia and eclampsia, the method of delivery must be tailored to each case. A team of obstetricians and intensive care unit specialists decided when the delivery should be done, and an anesthesiologist decided what type of anesthesia should be used. The delivery criteria should be based on gestational age at diagnosis (estimated fetal weight) and severity of preeclampsia. In general, both general and regional anesthetic procedures are equally suitable for cesarean delivery in pregnant women with severe preeclampsia if precautions are taken to establish a careful approach to either treatment [30]. Although it is an avoidable death, the mortality rate was high in our study, so it is mandatory to take action to reduce preeclampsia-related mortality and morbidity complications. The Millennium Development Goal 5 goal was to reduce maternal mortality by three-quarters between 1990 and 2015 [31]. Time is one of the main strategies to reduce maternal mortality, as the late decision to seek care, the late arrival at a health center, as well as the late offering of appropriate care, have major influences on maternal mortality [32].

The main factors that may increase mortality in our society include a lack of knowledge and awareness of pregnancy-related hypertension, a lack of antenatal care during pregnancy, and even an increased level of those who make deliveries in traditional nonhospital areas. Family planning, balanced nutritional status, increasing maternal knowledge and awareness, and taking the necessary prenatal care are factors that are required to reduce the occurrence of preeclampsia-related complications.

## 5. Conclusion

Our community has a high number of preeclampsia patients who require intensive care. Unfortunately, on the other hand, preeclampsia is highly associated with both maternal and child morbidity and mortality. Therefore, a guideline for severe preeclampsia and eclampsia must be made by the government and carried out at the regional level.

### 5.1. Limitations of Our Study

We focused on those who were admitted to the ICU; therefore, we may have missed those who died in the inpatient department before being transferred to the ICU. Again, since the study was retrospective in nature, there may be some unmeasured confounders. It is also hospital-based data, so it cannot be used for generalization. For a subsequent sizable multicentral investigation, these data serve as the foundational research.

## Figures and Tables

**Table 1:** Summary of sociodemographic factors for preeclampsia/eclampsia.

Sociodemographic		Frequency (%)
Gestational age	2^nd^ trimester	30 (42.3%)
	3^rd^ trimester	41 (57.7%)

No. of gravity	Primigravida	53 (74.6%)
	Multigravida	18 (25.4%)

No. of parity	Nulliparous	70 (%)
	Primipara	5 (7%)
	Multiparous	16 (23%)

Positive family history of G.H		11 (11.5%)

**Table 2:** Maternal complications during ICU.

Type Complications	Frequency (%)
Postpartum bleeding	36 (48%)
Severe anemia	9 (12.6%)
Stroke	8 (10.7%)
Peripartum infection	50 (66.7%)
Arrhythmia	57 (76%)
HEELP syndrome	16 (22.5%)
Acute renal failure	18 (25.4%)
Acute heart failure	6 (8.5%)
Placental abruption	1 (1%)

**Table 3:** Interventions during ICU admission.

Intervention type	Frequency (%)
Hemodialysis	10 (14.1%)
Mechanical ventilation	65 (91.5%)
Transfusion	51 (71.8%)
Central venous line	54 (76.1%)

## Data Availability

This research data will be available upon request (contact: drmohamedyalax@gmail.com).
